# Advanced model systems and tools for basic and translational human immunology

**DOI:** 10.1186/s13073-018-0584-8

**Published:** 2018-09-28

**Authors:** Lisa E. Wagar, Robert M. DiFazio, Mark M. Davis

**Affiliations:** 1 Department of Microbiology and Immunology, Stanford University School of Medicine, Stanford, CA 94305 USA; 2 Institute for Immunity, Transplantation, and Infection, Stanford University School of Medicine, Stanford, CA 94305 USA; 3 Howard Hughes Medical Institute, Stanford University School of Medicine, Stanford, CA 94305 USA

## Abstract

There are fundamental differences between humans and the animals we typically use to study the immune system. We have learned much from genetically manipulated and inbred animal models, but instances in which these findings have been successfully translated to human immunity have been rare. Embracing the genetic and environmental diversity of humans can tell us about the fundamental biology of immune cell types and the elasticity of the immune system. Although people are much more immunologically diverse than conventionally housed animal models, tools and technologies are now available that permit high-throughput analysis of human samples, including both blood and tissues, which will give us deep insights into human immunity in health and disease. As we gain a more detailed picture of the human immune system, we can build more sophisticated models to better reflect this complexity, both enabling the discovery of new immunological mechanisms and facilitating translation into the clinic.

## Background

Technological and reagent advances have accelerated our ability to perform human immunology research in a rigorous, ethical, and high-throughput manner. The goal of this review is to bring attention to the variety of techniques and options available for studying the human immune system directly and indirectly through judicious use of appropriate models, in vitro assays, and in vivo studies to improve the translatable potential of immunology research. Choosing the right model system for a given immunological question is essential. Much of what we currently know is derived from studies in inbred mice, and while they seem very similar to humans in many basic aspects, it appears likely that the much greater breadth of pathogen exposure experienced by human beings, together with their genetic heterogeneity, will result in many disparities. Intensive efforts over the past 30 years have seen the creation of ‘humanized’ mice with varying degrees of fidelity in accurately modeling human immune responses [1–5]. But the use of these animals presents a daunting problem for translation to humans because of the many incompatibilities between cytokines and cytokine receptors between species.

Although animal models certainly have a place in immunology research, it’s important to recognize their limitations in various aspects of recapitulating human immunity. Some human diseases have no appropriate animal model, and others are hampered by models that incompletely recapitulate key features of a human disease. Striking differences in the T cell responses of inbred mice cohabited with pet store mice have shown clearly that at least some of the differences between inbred mice and humans are due to environmental exposure [6], and even nonhuman primate models (NHP) have failed to predict human immune responses [7, 8]. Mice live one to two years, a miniscule amount of time compared to the average human lifespan. Not only is it virtually impossible to mimic a human lifetime’s worth of antigenic exposure in such a short period of time, but cell turnover is regulated in different ways in different species [9]. Most animal models are based around fixed genetic diversity (e.g., in major histocompatibility loci) and their distributions of immune-relevant receptors and ligands are quite distinct from those of humans (superbly summarized in [10]). If we seek to extend animal model findings to human immunity, it is important to get these genetic and cellular distributions right. Indeed, pre-clinical studies have often been poorly predictive of response in humans [11–17]. The combination of sophisticated animal models that are more relevant for studying human disease and our ability to perform direct ex vivo and in vitro high-throughput assays from human cohort samples suggests a bright future for understanding human immunity. Here, we discuss these new tools and systems that are available to better reflect the complexities of human immunity.

## Improved animal models

Owing to logistical and ethical considerations, experimental research in humans has limitations. This is especially the case for the testing of completely novel interventions and for mechanistic immunology research, although the tools that will overcome these challenges are being developed rapidly, as we discuss below. Therefore, animal models still have a place in the translational pipeline due to their ability to overcome these challenges. Myriad models are used for pre-clinical assessments, though generally speaking, the murine and NHP models are best for immunologic studies because of the availability of reagents and tools (Table 1). From small models (mice, guinea pigs, hamsters, zebra fish, and ferrets, among others) to large models (nonhuman primates, pigs, cows, sheep, and more), each model has its own set of advantages and disadvantages, and when choosing them, careful consideration should be made as to how they fit the research question. Here, we focus on the murine and NHP models and the recent advances in, and broad applications to, human translational immunology.

**Table 1:** An overview of animal models for translational studies for human immunology

Model system	Advantages	Disadvantages	Suggested model use	Translatability	Environmental factors
Conventional inbred, wildtype, and knock-out mice	• Consistency • Widely available • Cost • Easy to handle	• Poorly represent many human diseases	Fundamental immunology, pre-clinical work in some cases	+	Co-housing and diet may impact microbiota
Next-generation mice (humanized through genetic and/or tissue engineering)	• More potential for translation to humans • Many immunologic reagents • Easy to handle	• More expensive than conventional mice • Partial humanization means murine components can be confounding factors	Single-organ infections, such as liver cancer, especially metastasis; individual aspects of an immune response, such as Ig or HLA loci	++	Similar factors to conventional mice
Nonhuman primates (NHP)	• Human-like model • Many immunologic reagents • Limited MHC (certain species)	• Larger MHC than humans (most species) • Expensive • Ethical concerns	HIV, tuberculosis, many arthropod-borne viruses	++/+++	Typically not co-housed, but length in colony may impact response to perturbations
Other animal models	May model particular diseases more accurately than mice or NHP	Reagents limited or non-existent	Non-immunologic disease models, transmission studies (e.g., ferrets for influenza transmission studies recapitulate many features of human infection)	Up to +++	Many, as typically are not bred for research

Translatability (from weak (+) to strong (+++)) refers to the relative frequency of successfully identifying an immune phenomenon in the model system that closely mimics the relevant disease or condition in humans

*HLA* human leukocyte antigen, *Ig* immunoglobulin, *MHC* major histocompatibility complex

### Murine models

The advantages of mice are universally understood: they are small, tractable, inexpensive, and have many reagents readily available. Their tractability has led to many insights in basic immunology; many of the key insights gained in basic human immunology (such as lymphocyte receptor function, tissue homing, costimulation, and cytokine/chemokine signaling) were first elucidated in murine models. However, the inability of mice to mimic the human immune response means that they can be problematic in studies involving translation to the human system [11, 12, 18–21]. Many diseases that are of human relevance either do not exist or present differently in mice. For example, many viruses that cause disease in humans do not replicate in mice, and when they do, the pathology that results is often different to that observed in humans [22, 23]. Small molecules and other therapeutics can be species-specific and exert effects in humans that are different from those in animal models. Currently, the main tool to bridge this gap is the use of humanized mice.

Three of the most common types of murine models used for pre-clinical research are: genetically engineered mouse models; xenograft models, engrafted with either cell-line-derived (CDX) or patient-derived (PDX) tissue; and humanized models, which incorporate orthotopic implantations or injections and use tissue engineering and/or regenerative medicine approaches [24]. Humanized mice have been used for decades to model human immunity [25–28]. A breakthrough occurred in the early 2000s with IL2Rγ^null^ mice, which after engraftment are considered the most human-like model to date and encompass three main strains of mouse (detailed in [29]). There are a few approaches to engraft human immune cells into mice: using peripheral blood leukocytes (PBL); injection of severe combined immunodeficiency (SCID) reconstituting cells (SRC), also known as CD34+ hematopoietic stem cells (HSC); and the bone marrow/liver/thymus (BLT) model, established by transplantation of fetal liver and thymus and injection of autologous fetal liver HSCs [29]. The method of immune system engraftment [29] is important in relation to the research question being asked; for example, the BLT model would be most appropriate for human immunodeficiency virus (HIV)-related studies because it provides a higher level of engraftment of the human mucosal system [30]. The advantages and limitations of these models have been reviewed exhaustively by others [2, 29, 31–34]; therefore, in this review, we focus on a few recent advances.

Knock-in (KI) mice have emerged as a powerful tool to engraft whole parts of the human immune system, such as immunoglobulin (Ig) loci [35]. Transgenic human Ig loci were engrafted using bacterial artificial chromosome clones and sequential recombinase-mediated cassette exchange. This model has been utilized to study HIV humoral responses to novel interventions [36, 37] and is likely to be useful in any study in which humoral immune response is key (e.g., Zika or Dengue infection and disease). Advantages include being a controlled system and the maintenance of murine constant regions to avoid incompatibility effects, but this model does not reflect the other genetically diverse aspects of humans or their exposure history, nor does it reflect the immune system as a whole as other leukocyte populations remain murine. Another approach is to knock-in cytokines to enhance other immune responses, as has been achieved, for example, in IL-6 KI mice [5]. One new type of KI mouse (MISTRG) is developed using HSC engraftment on a background with multiple human cytokine knock-ins and demonstrates superior myeloid and natural killer (NK) cell development and hematopoiesis [3]. Human leukocyte antigen (HLA) transgenic mice have shown the ability to present human antigens in vivo in a model using human cytomegalovirus [38]. Engrafting humanized mice with umbilical cord blood is technically straightforward and provides T cells and autologous antigen-presenting cells (APCs) that can present cognate antigen [39]. The de novo transformation of B cells with Epstein-Barr virus (EBV) is observed in this model with tumor masses and tumor microenvironment similar to those observed in humans. Human bone marrow niche-forming cells can also be engrafted in the PDX model by either seeding the cells in vitro or by using a previously implanted scaffold. By using tissue engineering approaches to create a humanized microenvironment in addition to simply engrafting cells, one can study both hematopoiesis and malignancies in a more human-like system [40].

To further these ends, a framework has been proposed to generate a platform that would validate new humanized mice in a standardized manner; this approach merges tissue engineering and regenerative medicine techniques with benchmarks validated against human clinical data with known predictive power [24]. Others have proposed the co-engraftment of human tissues, for example, human HSC with human skin, liver, or lymph nodes to enhance effector and memory responses [41]. These murine models have translational potential for single-organ infections (e.g., hepatitis family viruses and human liver). One drawback to this system is that the model is not completely human, and the remaining murine cells and molecules may confound the interpretation. This could possibly be overcome by co-engraftment with multiple organs or humanization of multiple components, which then would increase the translational potential of this murine system.

### NHP models

At first glance, NHP models have several disadvantages compared to mice: they are large, expensive, less tractable, and involve ethical considerations. However, the immune system of NHPs more closely mimics that of humans, thus making them the most translational model system outside of humans themselves. NHPs have other advantages over mice. Some diseases can only be properly modeled in NHPs: for example, human HIV can only be modeled through simian immunodeficiency virus (SIV) and simian/human immunodeficiency virus (SHIV) because HIV cannot infect mice; and infecting mice with the causative agent of human tuberculosis (TB) disease neither causes clinical TB nor recapitulates TB pathology seen in humans, whereas NHP models (particularly the cynomolgus macaque) fully reflect both clinical TB and the disease pathology seen in humans [42, 43]. Although some diseases can be modeled in mice, their immune response may be totally different to that of NHP or humans, and could use immune mediators that may not exist in NHP or humans. Therefore, NHP have great translational value in pre-clinical studies.

NHP as an essential model for HIV have been well characterized with a plethora of experimental manipulations, including consideration of natural or hybrid challenge viruses, choice of NHP species, virus dose, challenge route, and more, all of which should be carefully considered during experimental design [44]. A cynomolgus macaque model of TB has been developed that fully recapitulates human TB, exhibits the full spectrum of clinical disease from latent TB infections to fulminant or septic TB, and has the range and types of pathology seen in humans [42, 43]. Novel frontline Ebola virus vaccines have been developed using the NHP model, because mice develop neither Ebola infection nor disease upon challenge [45, 46]. NHP have also been utilized to model many zoonotic viruses (*Flaviviridae*, *Togaviridae*, and others) [22] as well as influenza, although clinical influenza disease in NHP is still slightly different from that in humans [47]. Transplantation tolerance can also be modeled in NHP: a pilot in NHP demonstrated similar tolerance mechanisms to humans [48]. Aging and neurodegenerative diseases have been successfully modeled in NHP, which is a new avenue of interest as these diseases have been shown recently to have immunologic components and potential causes [49]. NHP grow old like humans: aging NHP and human brain transcriptomes are similar; NHP naturally display Alzheimer’s lesions such as amyloid plaques and aggregated hyperphosphorylated tau protein; and they display similar pathology from prion diseases [50]. As most diseases have some genetic component, the need for genetic characterization of NHPs has become apparent [51]. NHP genetics will aid in comparisons between NHP and human genomes, and finding and breeding natural variants will lead to the generation of specific disease models. NHP are outbred, so the impact of genetic background on specific genes or pathways can be measured in this system. Further development of NHP models through genome editing has been pursued [52, 53] but raises serious ethical considerations.

## Studying human immunity directly ex vivo and in vitro

**Fig. 1:**
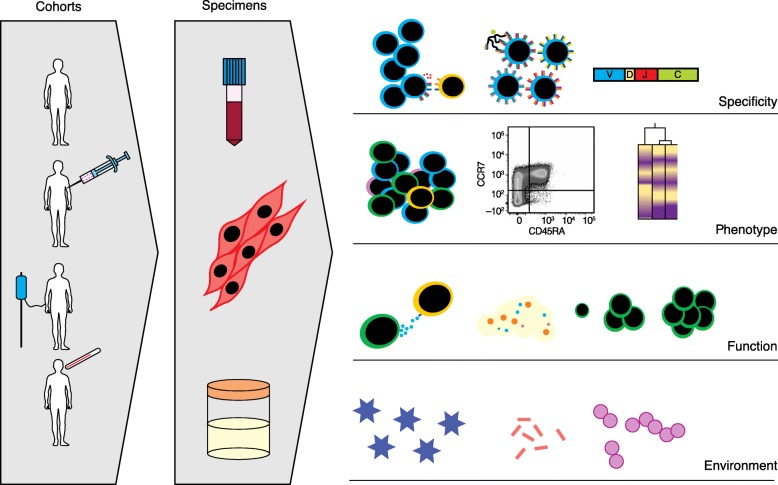
The wealth of human data for translational immunology. Consented cohorts of healthy donors and people in immune-perturbed conditions such as during illness, treatment, and immunization can provide insights into human immunity and disease-specific immune responses. Technologies now exist that allow us to study numerous sample types, including blood, tissue biopsies, saliva, urine, and feces, among others. Such samples are usually processed and banked, then run all together to limit batch variation. Depending on the questions to be answered, various assays can be run individually or in combination to gain insights into health or disease processes. These can include immune-cell-specificity assays (restimulation, tetramer staining, or repertoire analysis), broad phenotyping (flow and mass cytometry, RNAseq), functional readouts (cytotoxicity, metabolite detection, proliferation, or differentiation), or environmental contributions (microbiome or virome)

Given that there are many differences between the immune composition and function of humans and those of other animals, recognizing these disparities early on is crucial for translational purposes. One way to circumvent inter-species differences is to study human immune cells directly (Fig. 1). Most work has been (understandably) limited to blood, though discarded tissues and minimally invasive sampling have also been incredibly informative. Assessing tissues directly can be a resource for understanding cell types that do not circulate at high frequency (including resident memory, tissue-specific stroma, and germinal center populations) and in the study of immune infiltration in diseases with tissue- or organ-specific pathologies.

### Blood-based immunoassays

Peripheral blood has been used as a surrogate for the human immune system to study pressing immunological systems ranging from cell signaling to clinical trial outcome prediction. Indeed, peripheral blood studies are valuable due to the relative ease of sample acquisition, the low risk to the participant, and the potential for future translational applications in diagnostics and immunotherapeutics. Given that blood is the most frequent sample type used for immunology applications, there are numerous optimized assays for high-throughput analysis (Fig. 1). Peripheral blood has been used to provide information about the fundamental functions of immune cell types in humans [54–58]. Flow and mass cytometry are the tools of choice for establishing immune cell phenotypes and functions directly from ex vivo samples [59–65]. Adaptive immune repertoire analysis [66, 67] has also become prevalent, as discussed in detail below. Transcriptional and epigenetic profiling has revealed fundamental biological information about the regulation of immune genes and their contributions to human variation [68–72]. For example, Qu et al. [69] showed that sex has a strong impact on the regulome of CD4 T cells in healthy adults, and suggest that these differences may play an important modulatory role in autoimmune disease susceptibility. Standardized immunoassays [73–75], as well as more recent higher throughput techniques that have the potential to become standard assays [76–78], are widely available to measure circulating cytokines and other immune markers and metabolites in whole blood, plasma, sera, and culture supernatants for immune monitoring. These technologies can also be combined to provide an in-depth analysis of immune health and even to predict clinical outcome. For example, Lakshmikanth and colleagues [79] recently showed in a combination serum protein and mass cytometry phenotyping study of leukemia patients receiving allogeneic stem cell transplants that they could identify early immune features associated with patient outcome.

Whole blood and peripheral blood mononuclear cells (PBMCs) can be manipulated in vitro to study responses to a staggering diversity of self and non-self antigens, innate stimuli, and other molecules in health and disease [80, 81]. After in vitro PBMC stimulation with antigens of interest, specific T cells can be identified on the basis of dilution of an intracellular dye, indicating proliferation; cytokine production and capture upon restimulation can be measured with relevant peptides; and target cell killing or antigen specificity can be assayed using peptide:major histocompatibility complex (MHC) tetramers [82, 83]. Similarly, rare B cells can be detected from blood by staining or capturing cells through their specific B cell receptor using labeled or plate-bound antigens, respectively, in flow cytometry and ELISPOT experiments.

In human challenge models (HCMs), healthy people are intentionally infected with a controlled dose of a virus, bacterium, or parasite and then monitored typically through blood sampling (and/or mucosal sampling), sometimes while quarantined, for evidence of immune response and infection progression. These studies are often combined with vaccine efficacy testing or other interventions and post-infection treatment where appropriate. HCMs continue to be used to study human responses to influenza [84, 85] and other infectious diseases, including malaria [86, 87], dengue [88], hookworm [88], and numerous enteric infections such as *Salmonella typhi*, *Vibrio cholerae*, and *Escherichia coli* [89–94]. In a high-dose typhoid challenge model, one group showed that a large pre-infection population of functional, Typhi-responsive CD8 T cells in the blood was a good predictor of progression to typhoid disease. The authors proposed that the additional inflammatory response from a pre-existing pool of Typhi-reactive T cells might be sufficient to promote typhoid fever [89]. Interestingly, only subjects with the highest frequency of reactive CD8 T cells showed delay in disease development, suggesting that CD8 T cells play both a pathogenic and protective role during challenge.

At the same time, these models can tell us much about the immune features that are associated with resistance or susceptibility to infection, as well as the effectiveness of vaccines and treatments. HCMs are valuable because they allow vast quantities of detailed data to be collected from a closely studied cohort in a relatively controlled environment. As the typical population of interest consists of healthy adults, HCMs account for many aspects of human immunity that are absent in animal models, such as genetic variation, pre-existing immunological memory, environmental exposure, and the normal aging of the human immune system.

### Repertoire analysis

In recent years, substantial technological advancements and the reduced cost of high-throughput sequencing of T and B cell receptors have led to efforts to identify immune response signatures from sequence. Indeed, several groups have used T cell receptor (TCR) sequence analysis to study fundamental differences between T cell subsets (deeply from the repertoire of a single individual [95] and recently in combination with ATAC-seq (assay for transposase-accessible chromatin sequencing) [95], which allows both the TCR identity and DNA accessibility to be ascertained from individual cells) and the roles of T cells in the context of autoimmunity, cancer, and T cell pathologies [96–98] (Fig. 1). A recent study highlighted the value of TCR repertoire analysis in understanding the response to vaccination. Qi et al. [99] showed, in an elegant twin pair study of older individuals, that immunization with live attenuated varicella zoster virus (VZV) vaccine had numerous effects on the repertoire diversity of VZV-specific CD4 T cells. Overall, they found that diversity increased with immunization (with recruitment from the naïve T cell pool as well) and that although all VZV-specific clones expanded post-vaccination, they did not expand equally. On the basis of these findings, the authors proposed that although broadening the repertoire can have beneficial protective effects, the single immunization strategy employed here may not boost memory responses adequately.

Single cell sequencing [97] is becoming increasingly popular, as obtaining paired alpha and beta chain sequence data from TCRs of interest allows for recombinant expression and because yeast display libraries can be used to probe candidate ligands for TCRs of unknown specificity [100, 101]. Understanding an individual’s immune history and response to immune perturbation from TCR and B cell receptor (BCR) repertoire sequence alone would be transformative, but the incredible diversity of these receptors and the limited overlap between individuals even with the same HLAs and antigen exposure history creates a complicated analysis problem. However, recent advancements in TCR repertoire analysis tools that incorporate V gene usage and local motif searching techniques in the context of similar but non-identical (i.e., ‘convergent’) sequences suggest that, in the future, determining a TCR’s specificity from sequence alone could be possible [102, 103]. Similar strategies are being used for BCR repertoire analysis of similar, non-identical sequences to broaden our understanding of vaccine antigen targets for antibody responses [104, 105].

## Modeling immune tissues

Assays that use human tissues as the starting material are more likely to capture the essence of the immune microenvironment. Immune cells can have a relatively low frequency in the overall cellular composition of a tissue, and so studying relevant non-immune cells in concert with immune cells, particularly with pertinent cellular organization, can provide useful insights. We have started to learn a great deal about the tissue-resident immune distribution in human organs from recent studies of organ donors’ tissues [106].

Human PBMCs have also been used to successfully reflect some aspects of tissue-resident and lymph node biology in response to vaccine antigens. Using a system called human modular immune in vitro construct (MIMIC™), purified human T and B cells are combined with in vitro differentiated and antigen-pulsed dendritic cells to elicit antibody responses against vaccine candidates [107–109]. When compared to studies of unmanipulated PBMC cultures, these kinds of model systems show promise for improving vaccine efficacy predictions and for adjusting vaccine candidates prior to clinical trials. But overall, identifying predictive cellular biomarkers in peripheral blood for human vaccine responses and cancer immunotherapies, among many other areas, has been largely unsuccessful. Here, where the microenvironments and spatial organizations are unique, we believe that studying the relevant tissues can provide a clear advantage.

### Tissue-based immunity

For vaccine responses, the B cells responsible for forming a neutralizing antibody response are developed inside germinal centers (GCs) within lymphoid organs. Upon antigen arrival into a lymph node, T follicular helper cells (TFH) train GC B cells to form humoral responses. TFH and a variety of other cell types of hematopoietic and non-hematopoietic origin interact and deliver signals to GC B cells to promote survival, proliferation, affinity maturation, class switch recombination, and differentiation into memory B and plasma cells [110–114]. Most of these cellular processes are only briefly, or not at all, detectable in peripheral blood. Gathering information from human lymph nodes after antigen exposure can be problematic depending on node accessibility, size, and the extent of the response, although there have been some studies in which biopsies have been used to study lymph node-based responses [115, 116]. NHP studies have shown that analysis of lymph node fine needle aspirates can better predict neutralizing HIV *env* vaccine response [117, 118]. Two human studies, one in immunized healthy volunteers [119] and one in multiple sclerosis patients [120], have also shown that it is conceptually possible to study the accessible draining lymph nodes of immunized people. Given that fine needle aspiration is relatively non-invasive and considered a routine medical procedure for biopsy in cancer diagnoses [121], it seems plausible that future human immunization studies will incorporate this sampling strategy.

Similarly, peripheral blood studies have been largely unsuccessful in predicting therapeutic and prognostic indicators for cancer treatment, though this may be possible in some checkpoint blockade-treated cancers such as those treated with anti-PD-1 (anti-programmed death 1) [122]. Nevertheless, no currently approved tests use peripheral immune biomarkers to direct treatment [118, 123]. The tumor microenvironment and the associated immune infiltration has been much more informative in guiding treatment strategies [124–126]. In one study of metastatic melanoma patients treated with anti-CTLA-4 (cytotoxic T lymphocyte-associated protein 4; and later with anti-PD-1), early immune infiltration and activation at the tumor site were significantly correlated with treatment response [124]. The number and type of immune cells infiltrating the tumor site has been shown to have prognostic value [127, 128], warranting further investigation of immune recognition and function at tumor sites.

### Organoid-like culture

Organoids are in vitro representations of an organ or tissue that recapitulate the functional and structural features of the originating organ [129, 130]. Organoid culture has been used to model complex human and murine tissues, including lung, intestine, and brain [130, 131]. The use of the term 'organoid' varies substantially by field; although in many instances they are derived from an originating stem cell population, the consistent features of different organoid systems are relevant tissue patterning and retention of in vivo function. The organoid field has made significant advancements in modeling non-immune organs from mice and humans. Several groups have expanded organoid culture into immune tissues from mice that successfully support humoral responses [132–138]. Ankur Singh and colleagues extended organoid systems to immune tissues in a fully animal-independent way [132, 133]. Using an elegant murine cell-based system, they captured the essence of an immune microenvironment in vitro that permits B cell differentiation, promotes germinal center development, and supports antibody production [132, 133]. Although some facets of organoid culture are currently impractical to translate to a fully human system (dependence on exogenous protein expression from cell lines, re-introduction into living hosts), such methods have great potential to model immune processes. Our group has recently created human immune organoids from primary tonsil tissues that permit in vitro analysis of antigen-specific T and B cell responses. The system we have developed seeks to translate the existing excellent murine organoid models to humans and to allow more mechanistic immune studies to be performed on human tissues.

The organoid field has made substantial progress in modeling the tumor microenvironment and the corresponding tumor-infiltrating lymphocytes. A recent study identified features of treatment success or failure in response to checkpoint blockade using T cell-containing tumor spheroids [139]. These models are promising for providing a better understanding and potentially predicting patient response to checkpoint blockade prior to initiation of treatment in vivo.

## In vivo studies

The most physiologically relevant model of human immunity is the study of humans themselves in health and disease. Understanding immune variation among people can also tell us a great deal about how the immune system functions as a holistic unit during steady state and immune perturbations. Experiments dating back to just after the 1918 influenza pandemic indicate that people volunteered for infection challenge studies to improve understanding of disease transmission, immune memory, and the clinical course of infection [140–142]. Current human in vivo studies undergo rigorous ethics review and, for human challenge models in particular, health checkups prior to participation are part of the inclusion/exclusion assessment [143]. In vivo studies can tell us about the fundamental nature of immune cell functions, such as homeostatic proliferation and memory retention, that previously were almost exclusively studied in mice. For example, in a recent 10-year study of yellow fever vaccine recipients, Akondy et al. [144] determined that long-term persisting vaccine-specific CD8 T cells originate from early rapid dividers, subsequently divide less than once a year, and maintain a distinct transcriptional profile [144].

### Natural immune variation

There are insights to be gained from understanding human immune variation and so-called ‘experiments of nature’. Large-scale efforts have been undertaken in recent years to quantify genetic and environmental (e.g., pathogen exposure, immunization, chronic infection, microbiome, or maternal health) factors that contribute to observed immune variation among healthy people. The relative contributions seem to vary by cell type and human populations studied, as innate immune responses have been identified as more genetically controlled compared to adaptive responses [145–147]. Understanding immune variation has been a particularly rich area for HIV research too, with progress made in understanding immunological features of resistance against infection despite repeated exposure to the virus, long-term viral control, and non-progression to AIDS even in the absence of anti-retroviral drugs [148, 149].

Primary immunodeficiency patients who present with a constellation of susceptibility to infectious diseases and/or autoimmunity are also a window into the more mechanistic aspects of human immunity. In one recent clinical case, CD70 deficiency was shown to have a detrimental effect on T cell responses to EBV-infected B cells [150]. Izawa et al. [150] showed that disruption of the CD27/CD70 costimulation pathway resulted in defective T cell cytolytic function and proliferation against EBV-infected B cells through a TCR-mediated process. Reconstitution of CD70 expression restored normal functional activity. Individuals with these rare inborn mutations and their subsequent treatment have revealed a great deal about cell signaling in human immune cells and host–pathogen interactions in exquisite detail.

## In silico models and bioinformatics

Computational models for translational human immunology are often overlooked but useful tools. Computational power is now robust and sophisticated enough to model the complex processes of human immunity. This power is relatively cheap, easily reproducible, transparent, and high throughput, being able to perform hundreds or even thousands of ‘experiments’ in a single run. There are two main flavors of these tools*:* in silico models (or mechanistic models of immune processes); and bioinformatics (or data-driven models that link immune processes to clinical outcomes or other endpoints, such as biomarkers and predictive markers) [151].

In silico models incorporate available biological data into systems that can be tractable and high throughput. These biological data can come from small-scale individual studies or large-scale systems immunology projects. In silico models can integrate various types of data, including DNA, RNA, protein, metabolic products, and more. Common approaches to this type of mechanistic modeling include ordinary differential equations (ODE), agent-based models (ABM), and logical models [151]. These models can be deterministic (multiple runs will result in the same conclusion) or stochastic (probabilistic outcomes that may change after repeated runs) [151]. A recent example of the use of these models for translational human immunology is the utilization of molecular dynamics simulations in conjunction with models of affinity maturation to study the development of broadly neutralizing antibody production against HIV [152]. This study and many others from the same laboratory illustrate the power of integrating laboratory experiments, clinical data, computational inference, and mechanistic immune models [153]. Stochastic models have been developed that seek to mimic entire components of the immune system; these models are tunable and include both multi-scale (molecules, up to cells, up to tissues) and multi-compartment (in this case lung, blood, and lymph node) features [154]. These multi-scale, multi-component models have been used successfully to model TB granulomas, feature emergent behavior (i.e., placement of the TB causative agent into the lung model leads to granuloma formation), and are amenable to perturbations that would be impossible biologically (e.g., changes in local drug concentration, or finely tuning cytokine output from particular cells) [155]. In silico models have also been used successfully with cancer applications to model the tumor microenvironment in order to predict optimal anti-angiogenic therapies [156, 157] and to model tumors such as triple-negative breast cancer as a whole [158]. Models that simulate individual cells are currently in development [159]. These can then generate hypotheses that can be tested downstream through mechanistic studies in animal models (although the goal is to test them in human systems).

Bioinformatics relies on a bounty of available public data from a wide variety of sources. This data-driven approach is leveraged to identify biomarkers that can be validated biologically and to generate hypotheses about underlying immune mechanisms; it also serves as a source of carefully curated, publicly available data for comparison purposes [160, 161]. Genomics data, transcriptomics data, and many other -omics data that have been leveraged recently using bioinformatics approaches have been heavily reviewed and only brief examples will be given here. Using genomic data, genome-wide association studies (GWAS) can be performed that link features in DNA to disease traits. For example, a GWAS in patients with multiple sclerosis elucidated over 60% of genetic variation predicting multiple sclerotic onset, and this pattern largely overlapped with other autoimmune disease signatures [162]. Publicly available transcription data have likewise been used to generate predictive signatures for TB [163], systemic sclerosis [164], transplantation allograft survival [165], sepsis [166], anti-TNF (anti-tumor necrosis factor) treatment response in inflammatory bowel disease (IBD) patients [167], and many other conditions. Attention is increasingly turning to the epigenome, due to its role as a bridge between genetic regulation and the environment, and new technologies have recently emerged to probe this system, including ATAC-seq [69] and epigenetic CyTOF (EpiTOF) [168]. Many individual studies that produce these publicly available data are designed to eliminate heterogeneity, but these efforts can fail to exert predictive power and ignore the biological necessity (and relevance) of heterogeneity. One powerful technique that not only deals with but embraces heterogeneity is meta-analysis, which improves the reproducibility of bioinformatic models by integrating data from multiple studies [169]. Recent studies have also identified a bias toward richly annotated genes that should be avoided so that understudied genes, which may play key roles in disease, are not ignored [170]. The continued application and success of these -omics methods will generate more data that can be utilized in these types of models. Predictive markers can be tested in silico to reveal potential biological mechanisms, and in silico models can be used to identify putative predictive markers that can be validated with other approaches. Further development of these models is key to both expand the use of this technique and to even potentially disintermediate animal models.

## Conclusions

The future of translational human immunology is bright. Continuing to develop more sophisticated models of human immunity will improve our chances of successfully translating findings from fundamental biological studies to the clinic. Humanized mice can be a valuable and potentially translational model, but require further development for reliable and translatable data generation. The publication of negative (and positive) results for benchmarking will aid the development of this model. Developing mice with combined robust human memory B and T cell responses, neutralizing titers, and other immune features remains a major challenge [41]. Pet shop mice that are co-housed with inbred mice represent a novel solution to understanding some of the effects of diverse environmental exposure on immune cell functions [6, 171, 172]. These studies point to the fact that the immune system is finely tuned on the basis of microbial burden and that environment should be considered when attempting to model human immunity in the future. To be broadly useful, these environmentally exposed mice would need to be distributed by a central source. The judicious use of NHP to confirm the translational capabilities of studies before moving to people has clear benefits. That being said, some differences between NHP and humans could hamper translation; for example, MHC-I in NHP, which have multiple A loci and more than ten B loci, is much more complex than in humans [173].

**Fig. 2:**
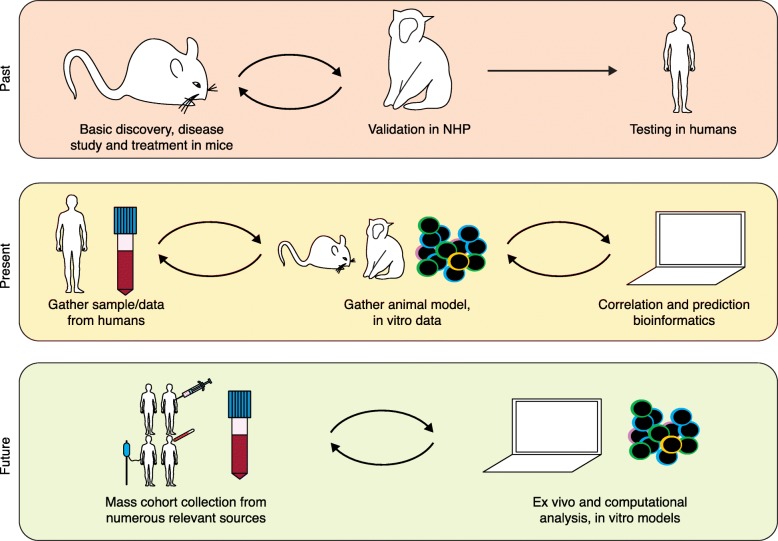
The shifting paradigm of translational human models. In the past, animal models were almost exclusively used for pre-clinical analyses, with limited success in translation to humans. NHP often served as a more relevant model for safety testing prior to attempts to test in humans, although on rare occasions this led to unanticipated and devastating effects in human trials. Currently, more strategies are incorporated into translational models, including sampling from people for in vitro assays. The data derived from human ex vivo and in vitro testing is often used to inform animal models and vice versa. As more high throughput data are made publicly available, computational models can contribute to the translational effort as well. In the future, it may be possible to bypass animal models entirely as more information is gathered from a variety of people of diverse health, genetic, and environmental backgrounds. As we gather broad data from human cohorts, our hope is that our predictive abilities and computational models will improve such that we no longer rely on animal models, although they will undoubtedly continue to play at least a supplemental role in translation

A main goal should be to continue to develop tools to close the animal gap, that is, to develop human-based systems that have the advantages of animal models, including high throughput and the potential for mechanistic, well-controlled studies (Fig. 2). Strong collaborations between scientists, engineers, and clinicians will help to accelerate this process. Along these lines, new approaches such as 'on-a-chip' assays continue to emerge. Microfluidics has been used to both capture immune cells for molecular analysis [174] and to model tissue organization and functions in which immune cells play an important role, including those in the gut, lung, blood vessels, and lymph nodes [175–177]. On-chip-based technologies have been a rich area of study for inflammation research, tumor–immune interactions, and early events of metastasis [178–180]. Nevertheless, there continue to be challenges for studying immune interactions with on-chip methods. There is a delicate balance in creating a model that captures the essence of the immune features to be studied that is, at the same time, not too reductive.

We are in an exciting time of human immunology during which high-throughput tools are increasingly accessible to study a wide array of immunological processes in humans. The growing availability of public data sets means that we should be using them more frequently in the hypothesis-generating process when embarking on new studies. At the same time, as a community we should strive to gather data from as diverse a population as possible so as to avoid over-extension from a single or small cohort.

## Abbreviations

ATAC-seq: Assay for transposase-accessible chromatin sequencing
BCR: B cell receptor
BLT: Bone marrow/liver/thymus
EBV: Epstein-Barr virus
GC: Germinal center
GWAS: Genome-wide association study
HCM: Human challenge model
HIV: Human immunodeficiency virus
HLA: Human leukocyte antigen
HSC: Hematopoietic stem cell
Ig: Immunoglobulin
KI: Knock-in
MHC: Major histocompatibility complex
NHP: Nonhuman primate
PBMC: Peripheral blood mononuclear cell
PD-1: programmed death 1
PDX: Patient-derived tissue
TB: Tuberculosis
TCR: T cell receptor
TFH: T follicular helper cell
VZV: Varicella zoster virus
